# Clinical and transcriptional signatures of human CD204 reveal an applicable marker for the protumor phenotype of tumor-associated macrophages in breast cancer

**DOI:** 10.18632/aging.102490

**Published:** 2019-12-04

**Authors:** Yunjie He, Siying Zhou, Fei Deng, Shujie Zhao, Wenquan Chen, Dandan Wang, Xiu Chen, Juncheng Hou, Jian Zhang, Wei Zhang, Li Ding, Jinhai Tang, Zuomin Zhou

**Affiliations:** 1 Department of General Surgery, The First Affiliated Hospital of Nanjing Medical University, Nanjing 210029, P.R. China; 2 The First Clinical Medical College, Nanjing University of Chinese Medicine, Nanjing 210023, P.R. China; 3 Department of Orthopedic, The First Affiliated Hospital of Nanjing Medical University, Nanjing 210019, P.R. China; 4 State Key Laboratory of Reproductive Medicine, Nanjing Medical University, Nanjing 210029, P.R. China

**Keywords:** CD204, breast cancer, tumor-associated macrophage, tumor immune microenvironment, subtypes of breast cancer

## Abstract

Background: Tumor-associated macrophages in human breast cancer are poorly understood. Specific tumor-associated macrophage-related molecular mechanisms among different intrinsic molecular subtypes remain unclear. Here, we have identified and explored the roles of the tumor-associated macrophages novel marker: CD204 in different subtypes of breast cancer.

Results: CD204 was upregulated in four subtypes of breast cancer, and this was associated with poor survival outcomes. CD204 could promote tumor cell proliferation, migration, and invasion and was involved in immune system-related pathways among all subtypes. Special pathways in each subtype were also found. High CD204 mRNA expressions were associated with high proportions of protumor immune cell populations, and most immunoinhibitors positive correlated with CD204 expression in all subtypes.

Conclusions: These findings contribute to a better understanding and managing the protumor phenotype of tumor-associated macrophages in different subtypes of breast cancer.

Methods: The expression of CD204 and its clinical outcome were analyzed. The roles of CD204 in the regulation of tumor cell proliferation, migration, and invasion were studied. Potential pathways influenced by CD204 were displayed. Immune cell infiltration in different CD204 mRNA expression status and correlations between CD204 and immunoinhibitors were also analyzed.

## INTRODUCTION

Breast cancer is the leading invasive malignancy and the second leading cause of tumor-related mortality among females worldwide [1]. Breast cancer has been recognized as a heterogeneous disease, and the following four intrinsic molecular subtypes have been identified using PAM50 gene signatures: luminal A, luminal B, HER2-enriched, and basal-like breast cancer [2]. Clinically, pathological classification based on immunohistochemical detection is mainly used instead [3]. Although different treatment modalities toward distinct subtypes are applied in clinical practice, there is a decrease in breast cancer mortality [4]. However, all advanced-stage breast cancers remain incurable, with a median overall survival (OS) of approximately 0.5–2.2 years [5]. Hence, it is essential to clarify the mechanisms specific to each of the molecular subtypes and exploit novel therapeutic options on this basis.

In recent years, tumor immune microenvironment (TIME) has been reported to play crucial roles in tumorigenesis, tumor progression, and metastasis [6]. Studies have shown that immunity and its interaction with tumor cells and TIME play divergent roles in different breast cancer subtypes. Generation of neoantigens is an important driver for immunogenicity in triple-negative breast cancer (TNBC) and HER2 amplified breast cancer. Moreover, HER2 itself can induce an oncogene addiction effect to facilitate a more tumor-supportive microenvironment among the HER2-positive subtype. Estrogen-mediated modulation of local inflammation in the microenvironment is found in hormone-receptor–positive but HER2-negative breast cancer [7]. However, more details about the different specific cellular components and molecular mechanisms among different intrinsic molecular subtypes of breast cancer remain unclear.

An increasing number of studies are being conducted with a focus on tumor-associated macrophages (TAMs) in recent years. TAMs constitute over 50% of the number of cells in breast malignancies [8]. In mouse models, most TAMs in breast cancer microenvironment behave as the M2-like phenotype, with protumor characteristics [9]; for example, a positive feedback loop between basal-like mesenchymal cancer cells and TAMs is identified as essential for the metastasis of breast cancer among mice [10]. In a mouse model of HER2-positive breast cancer, TAMs were found to orchestrate breast cancer dissemination during very early stages by dismantling E-cadherin junctions, ultimately fueling breast cancer progression and late stage metastasis [11]. In addition, insulin-like growth factors 1 and 2 are highly expressed on TAMs; their blockade increased the paclitaxel efficacy in a the mouse model of metastatic breast cancer [12]. The protumor behavior of TAMs in mouse models has made them attractive therapeutic targets. Approaches include inhibition of TAMs recruited to tumor cells, functional re-education of TAMs to an anticancer mode by switching them into an M1-like phenotype, and induction of tumor cell phagocytosis [13–18]. Several drugs targeting CSF1-CSF1R, CD47-SIRPα, CCL2-CCR2, Ang2-TIE2, CD40, CR3, and TLR7 are employed in clinical trials [19]. However, these drugs showed little efficacy. A probable reason for this is that these therapies are limited by the lack of TAM-specific markers and little is known about TAMs in humans [20, 21].

In this study, to explore the representative characters of TAMs among subtypes of human breast cancer in depth, we focused on studying protumor TAM markers among human breast cancer tissues: CD163, CD204, CD206, which are commonly used in mouse models [22]. We described the various expression patterns of CD204 and the clinical prognosis outcome. We also investigated the roles of CD204 plays in the regulation of tumor cell proliferation, migration, and invasion. GSEA (Gene Set Enrichment Analysis) in the TCGA databases revealed common and special pathways, which were in enriched in high-CD204 expression patients of the four subtypes. We further evaluated the infiltration of specific immune cells in patients with different levels of CD204 expression and also demonstrated the correlations between CD204 and immunoinhibitors among the TIMEs of breast cancer. This comprehensive integrated research was conducted to systematically identify the potential roles of CD204 and novel pathways influenced by CD204 among breast cancer. We demonstrated that the clinical applications of CD204 could be explored further.

## RESULTS

### Expression patterns of CD204 among different molecular subtypes at different clinical stages

**Figure 1:**
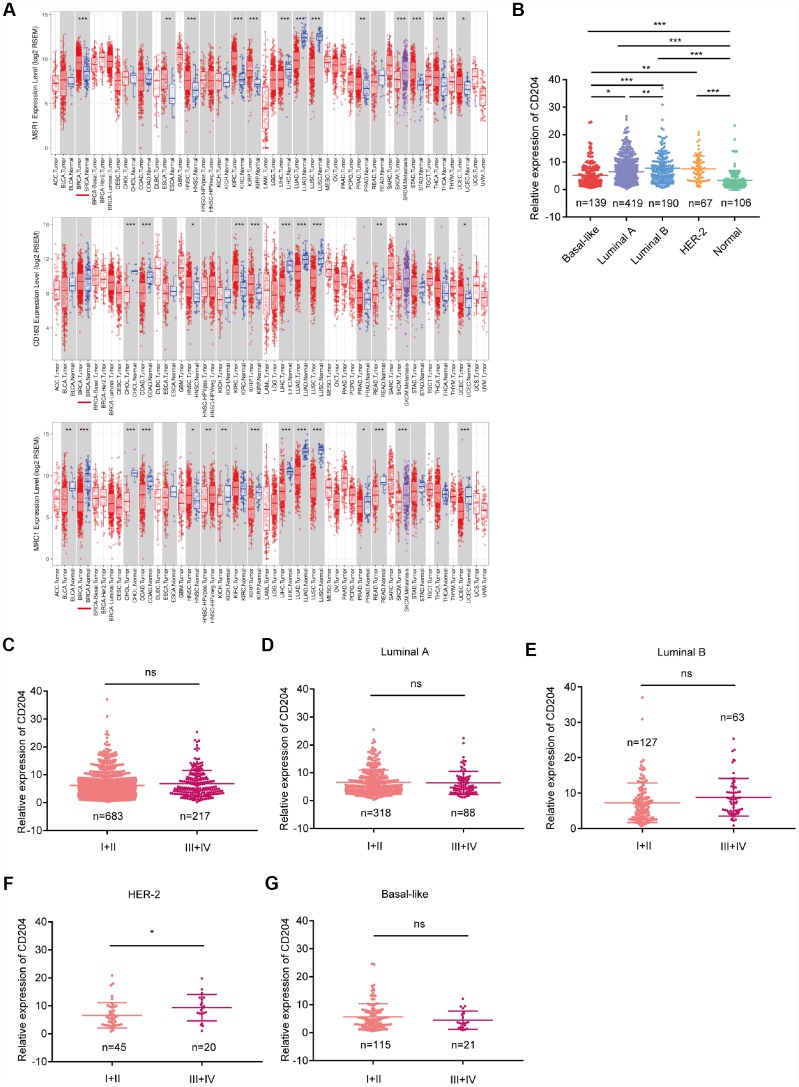
Comparison of CD204, CD163, and CD206 mRNA expressions between tumors and normal tissues (**A**) comparison of CD204 mRNA expression between different subtypes (**B**) of all cases of breast cancer between stage I-II and III-IV (**C**), and in each subtype, including luminal A (**D**), luminal B (**E**), HER2-positive (**F**), and basal-like (**G**) cancers.

CD68, a pan-macrophage marker, is most extensively used to detect TAMs; however, the expression of CD68 in cancer and stromal cells was found. Therefore, the use of this marker to identify TAMs necessitates carefully evaluation [23]. Recently, CD163, CD204 (MSR1), and CD206 (MRC1) have been used to detect TAMs in mouse models [22]. To determine how these markers are expressed in tumors in comparison with normal tissue, the mRNA levels were analyzed using TCGA database. Compared with normal tissue, only CD204 was to be upregulated in breast cancer tissue (Figure 1A), suggesting its important role. Thus, we selected CD204 for further investigation. Different CD204 expression conditions among subtypes were also identified (Figure 1B). However, we did not find any differences in the CD204 expression levels among samples obtained from patients with clinical stage I-II and III-IV breast cancer (Figure 1C). We further analyzed the CD204 expression levels in each subtype at different clinical stages. Only in the HER-2-enriched subtype, CD204 expression was higher in stage III-IV than in stage I-II (Figure 1D–1G), further suggesting the diverse roles of CD204 in different breast cancer subtypes.

### Clinical outcome of CD204 among different molecular subtypes

**Figure 2:**
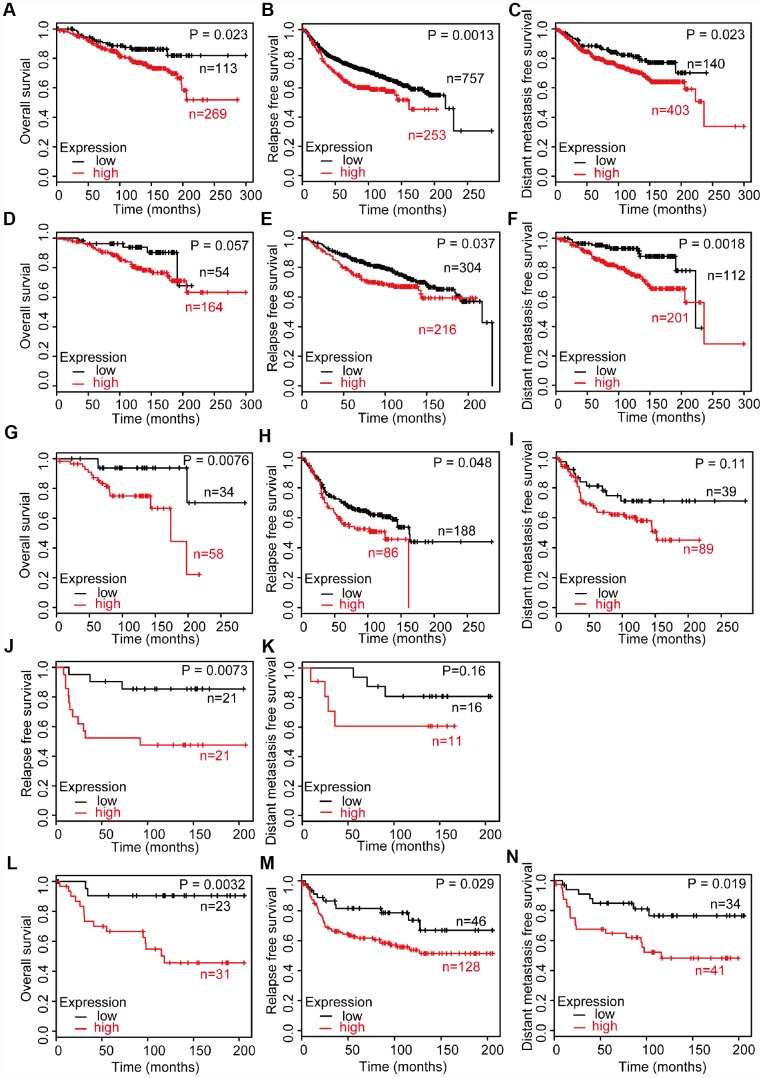
Survival analysis of breast cancer with high and low CD204 expression statuses among untreated patients, including all cases of breast cancer (**A**–**C**), luminal A (**D**–**F**), luminal B (**G**–**I**), HER2-positive (**J**, **K**), and basal-like (**L**–**N**) subtype.

Then, we investigated the prognosis according to the CD204 expression levels among all untreated breast cancers and the four molecular subtypes using GEO datasets. GEO datasets contain a larger sample size (patient total number, 3951) than the TCGA database (patient total number, 815) and own systemically untreated patients’ cohorts that are appropriate for the clinical outcome analysis of TAM-related markers [22, 24]. Because macrophages influence the prognosis of breast cancer after chemotherapy and other treatment, overall survival (OS), relapse free survival (RFS), and distant metastasis survival (DMFS) were analyzed. Among all breast cancers, patients with lower CD204 expression levels have better OS, RFS, and DMFS (Figure 2A–2C). Better OS, RFS, and DMFS were also found in lower CD204 expression group among the luminal A subtype (Figure 2D–2F). Subsequently, better OS and RFS were shown in luminal B patients with lower CD204 expression levels (Figure 2G, 2H). In the HER2-enriched subtype, better OS was found in lower CD204 expression group (Figure 2J) and better OS, RFS, and DMFS were shown in basal-like subtype with a lower CD204 expression level (Figure 2L–2N). In addition, there was no significant difference in DMFS between higher and lower CD204 expression groups in luminal B and HER2-amplified subtype (Figure 2I, 2K), which may partly be the result of the smaller sample size. OS analysis of HER2-amplified subtype was not performed owing to the low number of cases. Potential results of better DMFS in luminal B and HER2-amplified subtype with a lower CD204 expression level were observed. Generally, high CD204 expression levels in all subtypes demonstrated poor clinical outcomes in patients with breast cancer.

### CD204^+^ tumor-associated macrophages promote proliferation of breast cancer cells

**Figure 3:**
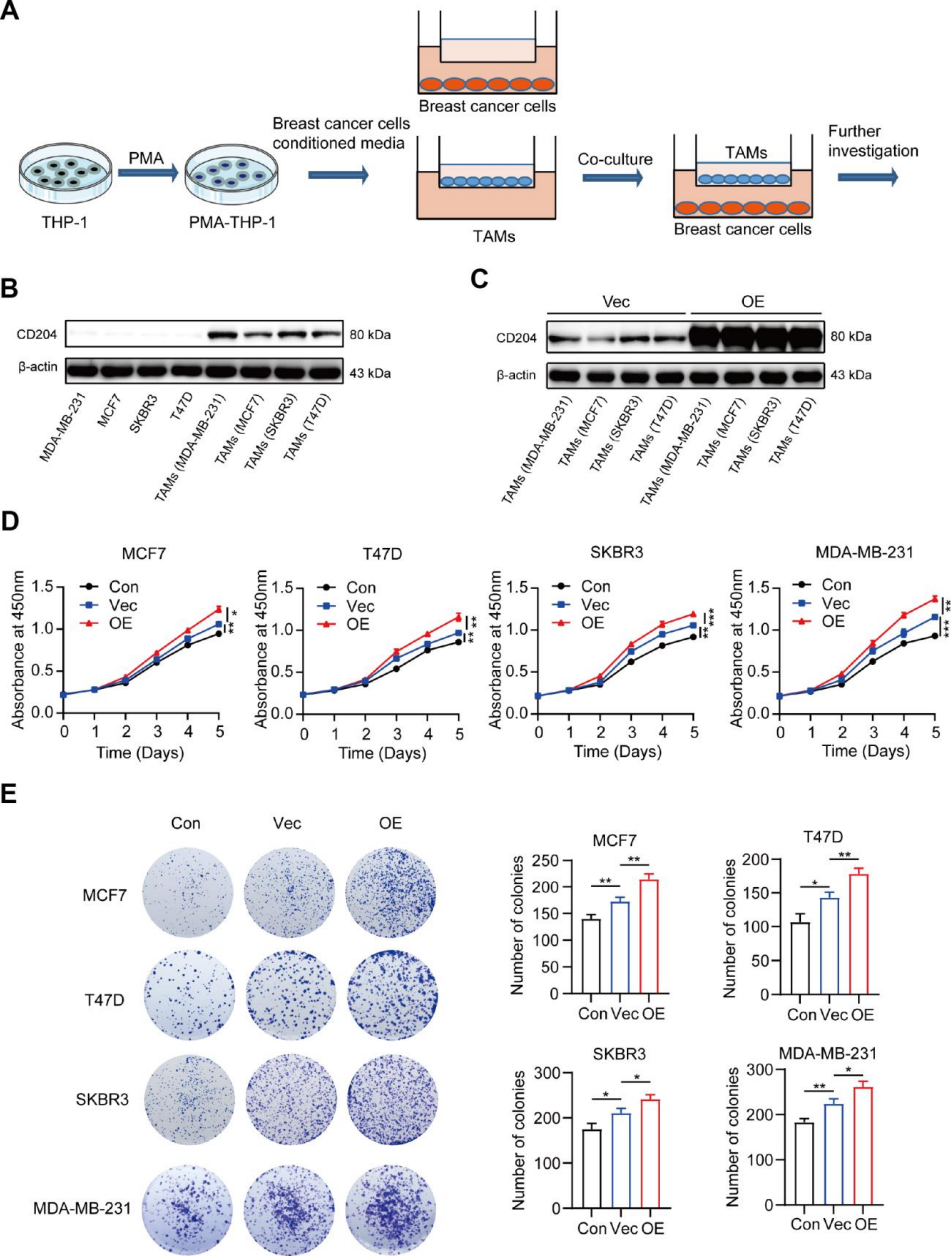
Schema for representing the experiment procedures (**A**). Protein expression of CD204 was detected by Western blotting (**B**, **C**). Proliferation of breast cancer cells alone (Con) or co-cultured with macrophages transfected with negative control plasmid (Vec) or macrophages transfected with CD204 plasmid (OE) was assessed by CCK-8 and colony formation assays (**D**, **E**).

To investigate the roles CD204 play in the regulation of tumor cell proliferation, we utilized an *in vitro* model of TAMs (Figure 3A). The human monocyte cell line THP-1 was induced into macrophages by treatment with PMA for 24 h and then cultured with conditioned media from four different breast cancer cell lines (MCF7, T47D, SKBR3, and MDA-MB-231). Based on the previous literature, the luminal A subtype comprised MCF7 cells, the luminal B subtype comprised T47D cells, the HER-2-enriched subtype comprised SKBR3 cells, and the basal-like subtype comprised MDA-MB-231 cells [25]. We found that CD204 was only expressed in TAMs rather than breast cancer cells (Figure 3B). Next, we successfully increased the CD204 expression level in TAMs and defined these as CD204^+^ TAMs (Figure 3C). Moreover, CD204^+^ TAMs could promote proliferation of cells of all subtypes of breast cancer, as assessed by CCK-8 and colony formation assays (Figure 3D and 3E).

### CD204^+^ tumor-associated macrophages enhance migration and invasion of breast cancer cells

**Figure 4:**
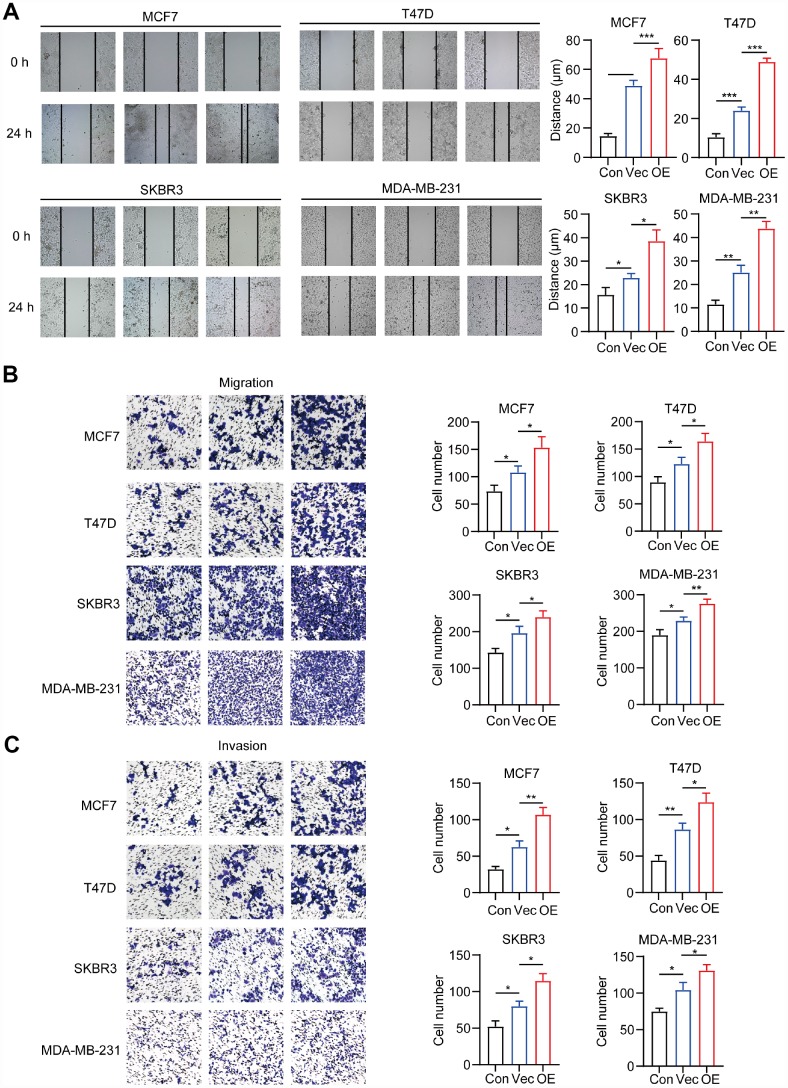
Migration and invasion capacity of breast cancer cells alone (Con) or co-cultured with macrophages transfected with negative control plasmid (Vec) or macrophages transfected with CD204 plasmid (OE) was determined by the wound healing assay (**A**) and transwell coculture system (**B**, **C**).

We further explored the effects of CD204^+^ in breast cancer migration and invasion. CD204^+^ TAMs significantly increased the wound-healing ability of all breast cancer subtypes (Figure 4A) and enhanced their migratory and invasive capabilities (Figure 4B and 4C). Altogether, these results suggested that all breast cancer subtypes upregulated the expression of CD204 in TAMs, and CD204^+^ TAMs improved the malignant abilities of breast cancer cells *in vitro*.

### Common KEGG and HALLMARK pathways influenced by CD204 in breast cancer cells

**Figure 5:**
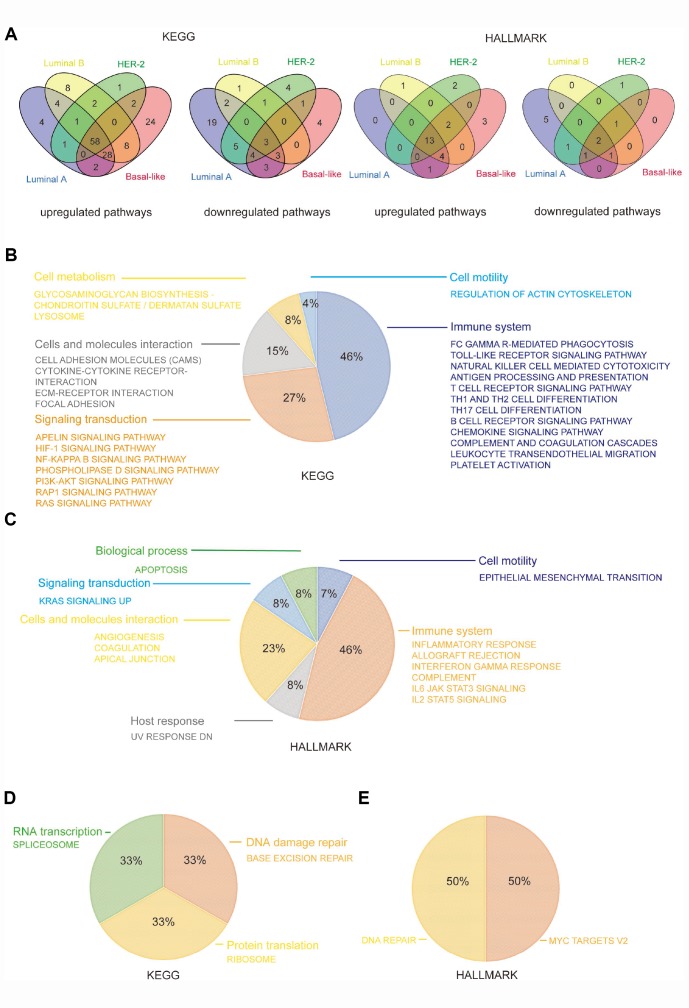
**CD204 related common KEGG and HALLMARK pathways among the four subtypes of breast cancer.** Venn diagram depicting the upregulated and downregulated pathways (**A**). Constitution of common upregulated KEGG pathways (**B**). Constitution of common upregulated HALLMARK pathways (**C**). Constitution of common downregulated KEGG and HALLMARK pathways (**D**).

To further explore the mechanism involved in the promotion of breast cancer by CD204, we performed GSEA to identify the KEGG (Kyoto Encyclopedia of Genes and Genomes) and HALLMARK pathways with which significantly upregulated or downregulated gene sets correspond. Among the KEGG pathways, we found that the four subtypes had 58 upregulated gene sets and three downregulated gene sets in common. Among the HALLMARK pathways, we identified 13 upregulated gene sets and 2 downregulated gene sets shared by the four subtypes (Figure 5A).

We found that common upregulated KEGG pathways, such as phagocytosis; the toll-like receptor signaling pathway; natural killer cells, T cells, B cells, and antigen presentation pathways; chemokine and complement cascades; leukocyte migration; and platelet activation, were primarily involved in the functions of the immune system (46%), including innate and adaptive immunity. Several cell signaling transduction pathways accounted for 27%, including apelin, HIF-1, NF-kB, phospholipase D, PI3k–Akt, RAP1, and Ras signaling pathways. Cell adhesion molecules, cytokine and ECM–receptor interaction, and focal adhesion were also identified as interaction pathways in cells and molecules (15%). Moreover, cell metabolism, including glycosaminoglycan and lysosome pathways (8%), and cell motility pathway, including actin cytoskeleton regulation (4%), were also influenced by CD204 in all four subtypes (Figure 5B).

Similar to KEGG pathways, we found that common upregulated HALLMARK pathways were primarily involved in functions of the immune system (46%), including inflammatory response, allograft rejection, interferon gamma response, complement, IL6-JAK-STAT3 signaling, and IL2-STAT5 signaling pathways. Cellular and molecular interaction (23%), signaling transduction (8%), biological process [apoptosis (8%)], and cell motility [epithelial mesenchymal transition (7%)] were also shown to be regulated by CD204 (Figure 5C).

In addition, among three commonly downregulated KEGG pathways, one was involved in RNA transcription (33%), one in protein translation (33%), and one in the DNA repair (33%). DNA repair was also shown to be influenced in common downregulated HALLMARK pathways (Figure 5D and 5E). These results further confirmed that CD204 plays a crucial role in the management of the immune system and tumor malignancy-related pathways in breast cancer cells.

### Special KEGG and HALLMARK pathways influenced by CD204 in four breast cancer subtype cells

**Figure 6:**
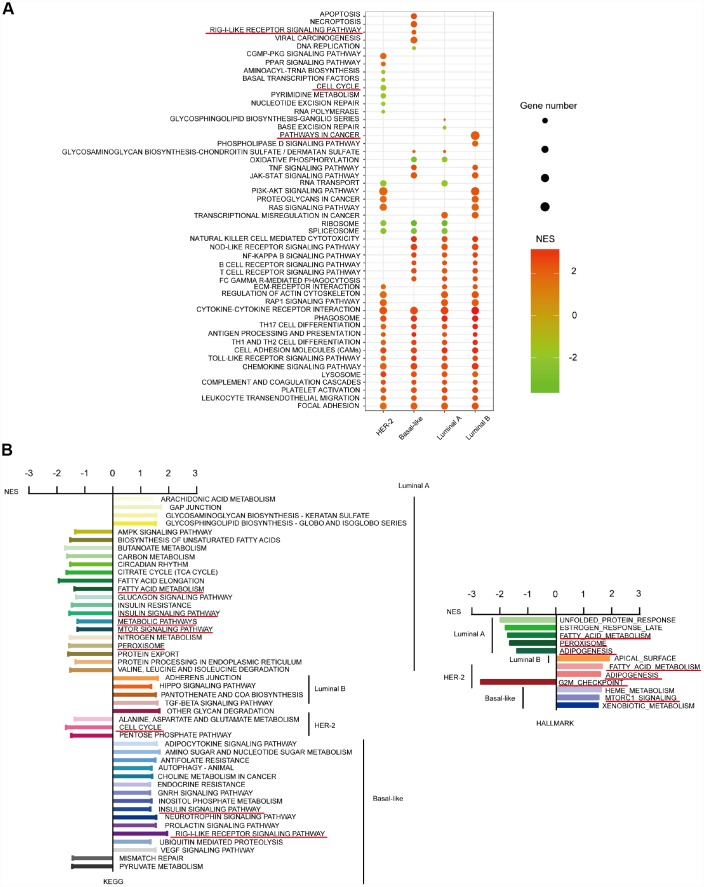
**CD204-related special KEGG and HALLMARK pathways for the four subtypes of breast cancer.** Top 30 gene sets and corresponding KEGG pathways influenced by CD204 between four subtypes using NES score and gene number screening (**A**). Special pathways in each subtypes (**B**).

We further studied the differences in the top 30 KEGG pathways influenced by CD204 among the four subtype cells using NES scores and gene number screening. Each subtype had a specific pattern (Figure 6A). Special KEGG and HALLMARK pathways influenced by CD204 in subtype cells are shown in Figure 6B. We determined that considerable innate and adaptive immune pathways and cell interaction pathways in the four subtype cells were significantly affected by CD204. Otherwise, in the luminal A subtype cell, upregulated or downregulated special gene sets were involved in tumor-related metabolic pathways, including the TCA cycle, fatty acid metabolism, nitrogen metabolism, peroxisome, mTOR, and AMPK signaling pathways. In the luminal B subtype cells, the pathways were significantly influenced by CD204. Hippo and TGF-beta signaling pathways were found to be unique, suggesting important roles of CD204-related activities. In the HER2 subtype cell, cell cycle-related pathways were found to be among the top 30 KEGG or special pathways. In the basal-like subtype cell, RIG-I-like receptor pathways were found to be significantly and uniquely influenced by CD204. Interestingly, downregulated gene sets of fatty acid metabolism and adipogenesis were found in the luminal A subtype, whereas upregulated gene sets of those were shown in the HER-2-enriched subtype. Downregulated gene sets of insulin and mTOR signaling pathways were also found in the luminal A subtype, whereas upregulated gene sets of those were displayed in the basal-like subtype cell. This further suggests that CD204 plays different roles in each breast cancer subtype. The detailed and representative special pathways influenced by CD204 are shown in Supplementary Figure 1.

### Immune cell infiltration in different CD204 mRNA expression status and correlations between CD204 and immunoinhibitors

**Figure 7:**
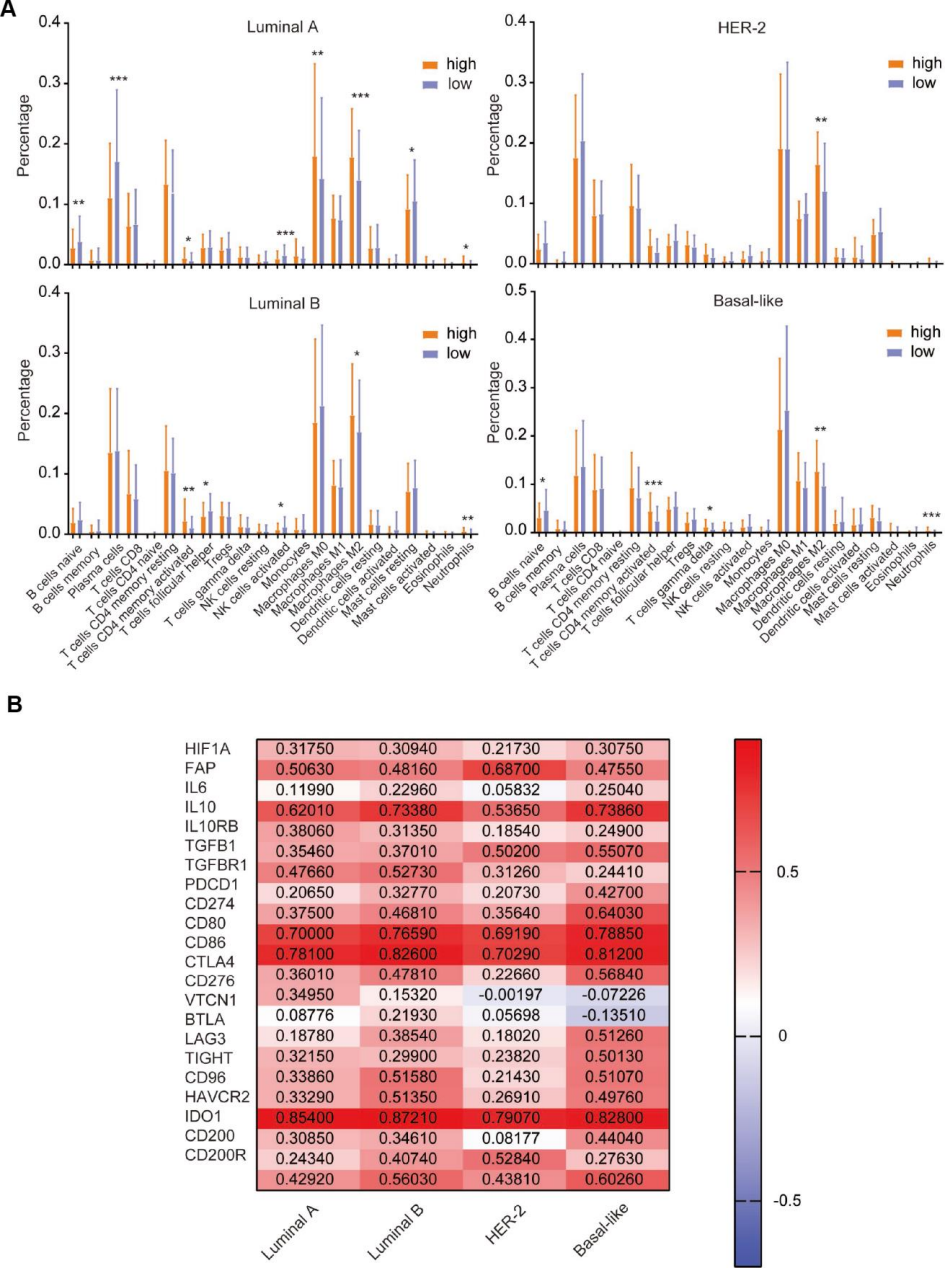
Comparison of the expression of LM22 identified by CIBERSORT between high and low CD204 expression groups (**A**). Correlation of CD204 and immunosuppressive molecules among the luminal, HER2-positive, and basal-like subtypes of breast cancer (**B**).

Previously, we found that CD204 significantly influenced the immune system in all four subtypes of breast cancer. To further investigate the infiltration of specific immune cells in different CD204 expression statuses, we used CIBERSORT to calculate the relative proportion of 22 types of immune cells recognized as LM22 in the TCGA database, as shown in Figure 7A. High CD204 mRNA expressions were associated with high proportions of M2 macrophages in all four subtypes, further suggesting that CD204 is a marker for the protumor phenotype of TAMs in breast cancer cells. Moreover, in the luminal A subtype, high proportions of M0 macrophages, neutrophils, and CD4 memory-activated T cells and low proportions of naïve B cells, plasma cells, activated NK cells, and resting mast cells were observed among patients with high CD204 expression levels. In the luminal B subtype, high proportions of CD4 memory-activated T cells and neutrophils and low proportions of follicular helper T cells and activated NK cells were found among patients with a high CD204 expression level. In the basal-like subtype, higher proportions of CD4 memory-activated T cells, γδT cells, and neutrophils and a lower proportion of naïve B cells were shown in patients with a higher CD204 expression level. Neutrophils and γδT cells could promote breast cancer metastasis [26, 27]. CD4 memory-activated T cells facilitated tumor growth [28], whereas B cells, plasma cells, and NK cells inhibited tumor growth in breast cancer [29]. In addition, follicular helper T cells predicted good prognoses of breast cancer patients [30]. Thus, we revealed the fact that in breast cancer, tumors with high-CD204 mRNA had higher rates of protumor immune cell infiltration and lower rates of antitumor cell infiltration.

Cumulative evidence demonstrates that the protumor phenotype of TAMs often induces immunosuppression in the TIME [31, 32]. To further investigate the role of CD204 involved in the TIME of breast cancer, we first explored the correlations between CD204 and TIME-related immunoinhibitors among luminal A, B, HER-2-enriched, and basal-like subtypes (Figure 7B). There were significant positive correlations (r > 0.2) between the expression of CD204 and hypoxia marker HIF1A; cancer-associated fibroblast marker FAP; tumor immunosuppressive cytokines IL10, TGFB1, and TGFBR1; and multiple immunosuppressive checkpoints PDCD1/CD274, CD80, 86/CTLA4, LAG3, TIGHT, CD96, HAVCR2, and CD200/CD200R in all subtypes. Positive correlations were also observed between CD204 and IL6 in luminal B and basal-like subtypes between CD204 and IL10RB in the luminal and basal-like subtypes, between CD204 and VTCN1 in the luminal B subtype, between CD204 and CD276 in the luminal A subtype, between CD204 and BTLA in the luminal B and basal-like subtypes, and between CD204 and IDO1 in the luminal and basal-like subtypes but not in the HER-2-enriched subtype. Therefore, we found that CD204 was positively correlated with most TIME-related immunosuppressive molecules. Altogether, these results reveal the potential importance of CD204 in the TIME of breast cancer.

## DISCUSSION

In the past five years, cancer immunotherapy has achieved great success with respect to lung cancer, melanoma, and other hematological malignancies treatments, especially via the PD-L1 and PD-1 antibodies. However, breast cancer is recognized as a cold tumor because the immune cytolytic activity toward immunotherapy, burden of nonsynonymous tumor mutations, and predicted load of neo-epitopes-factors linked to response to checkpoint blockade are all relatively modest [33]. A breakthrough from the Impassion130 clinical trial was reported at the 2018 European Society for Medical Oncology Congress in the breast cancer immunotherapy area. The PD-L1 antibody atezolizumab plus chemotherapy (nab- paclitaxel) combination therapy was found to prolong progression-free survival in patients with metastatic TNBC in both intention-to-treat population and the PD-L1–positive subgroup and to significantly increase OS in patients with PD-L1–positive tumors [34]. The benefit with anti–PD-L1 treatment provided evidence of the efficacy of immunotherapy in breast cancer patients. Recently, therapeutic strategies to TIME of breast cancer including efforts to (1) expand CD8+ T cells, NK cells, and DCs, (2) improve antigen presentation and (3) decrease inhibitory cytokines, TAMs, regulatory T cells and myeloid derived suppressor cells (MDSCs) had shed light on altering the TIME to make breast tumors more sensitive to immunotherapy [35]. Thus, deeply understanding the biological functions and molecular mechanisms of immune or TIME of breast cancer is necessary for investigating more effective therapy strategies.

CD204, encoded by gene macrophage scavenger receptor 1 (MSR1), also known as the scavenger receptor A (SR-A), is the class A scavenger receptor with homotrimeric transmembrane structures. CD204 is preferentially expressed on mouse and human macrophages and implicated in many macrophage-associated physiological and pathological processes [36, 37]. Furthermore, recently CD204 is found overexpressed on TAMs, which are key components of the TIME. Studies have also displayed that co-culturing of macrophages with murine ovarian and pancreatic cancer cells upregulates macrophage CD204 expression, and TAMs of a high CD204 expression level have the ability to promote tumor progression *in vitro* and *in vivo* [38]. A high CD204-positive TAM density in human is also associated with poor prognosis in various tumors [39–42], including breast cancer [32, 43]. In CD204-deficient mice, macrophages showed antitumor activity in EL4 lymphoma by upregulating nitric oxide and interferon-γ [44], demonstrating the important roles of CD204. Thus, CD204 represents a novel marker of TAMs and a target for tumor therapy toward the microenvironment.

In this study, we found that the CD204 expression levels differed among the four subtypes of breast cancer but were all higher than the CD204 expression levels in normal tissue. The highest expression of CD204 was found in the luminal B subtype. Yuko Miyasato et al. used immunohistochemistry to detect protein expression and found that the density of CD204-positive TAMs was higher than that of CD163-positive macrophages in 149 breast cancer tissues in addition to being significantly related to triple-negative cancer cells and the Ki-67 index [32]; this also indicates the importance of CD204 in different subtypes of breast cancer. A larger sample size is required to further detect CD204 protein expression among clinical tissue. We also found that a higher CD204 expression level indicated worse clinical outcomes among almost all patients. These results suggest that CD204-positive TAMs are predictive of the prognoses. It is necessary to further explore this potential predictive ability to create therapies to which breast cancer patients respond.

Our results also demonstrated the specific molecular mechanisms of CD204. CD204 participated primarily in the immune system, and the specific pathways differed among the four subtypes. Intriguingly, gene sets from lipid metabolism-related pathways showed adverse NES scores between the luminal A or B and HER-2-enriched or basal-like subtypes, suggesting pathways with different activation or inactivation states.

By further comparing the immunocytes’ infiltration between high and low CD204 groups, we revealed that tumors with high CD204 mRNA levels had higher protumor M2 macrophages in all subtypes. In addition, they had lower antitumor plasma and resting mast cells in the luminal A subtype, activated NK cells in luminal A and B subtypes, and follicular helper T cells in the luminal B subtype [30, 45, 46]. These results suggest that in breast cancer, CD204 influences the infiltration of immunocytes in different subtypes.

In addition, most TIME-related immunosuppressive molecules, including HIF1A, FAP, IL10, TGFB1, and multiple checkpoint inhibitors positively correlated with the CD204 expression levels in all subtypes; however, IL6, CD276, VTCN1, and IDO1 displayed different correlation values in different subtypes, providing potential evidence for different combination strategies of breast cancer immunotherapy in the four molecular subtypes.

Recently, another molecule: Ionized calcium-binding adaptor molecule 1 (Iba-1) has been used as a macrophage marker in human and mouse studies [47, 48]. Iba-1 was often defined as a maker of microglia/macrophage within central nervous system microenvironment [49, 50]. Other researches also used Iba-1 as a pan-macrophage marker in undifferentiated sarcoma and clear cell renal cell carcinoma [51, 52]. Based on literatures, the specific function of Iba-1 positive macrophages in breast cancer is largely unknown, and it is worthy to be studied in the future.

In summary, our results demonstrated the clinical and transcriptional landscape of CD204 in four subtypes of breast cancer. We demonstrated that CD204 could become an applicable TAM marker in human breast cancer.

## MATERIALS AND METHODS

### Expression profiles analysis

The CD163, CD204, and CD206 mRNA expression profiles among cancers were analyzed using the online tool TIMER [53]. The CD204 mRNA expression profiles and relative clinical data among the four breast cancer subtypes and normal breast tissues were downloaded from TCGA database. (https://tcga-data.nci.nih.gov/). Samples with equivocal information were filtered out. Clinicopathological characteristics, including PAM50 subtypes, TNM stage, and histological type were collected.

### Survival outcome analysis

The effect of CD204 expression on survival outcomes, including OS, RFS, and DMFS, of patients with breast cancer were analyzed using the GEO datasets on the Kaplan–Meier plotter online software (http://kmplot.com/analysis/) [54]. Systemically untreated patients were split by auto-select best cutoff. The follow-up threshold was all along. A probability value (p) of <0.05 was considered to be statistically significant.

### KEGG/HALLMARK pathways analysis using GSEA

GSEA (http://www.broad.mit.edu/gsea/) was performed [55, 56] to identify gene set changes with high CD204 expression vs. low CD204 expression groups that were divided by the median value among four subtypes using expression values from the TCGA database. Genes were ranked according to their fold change in high vs. low CD204 groups, and an enrichment score (ES) ranging from −1 to 1 was computed. High ES values correspond to an enrichment of the reference class among genes that are upregulated, whereas low ES values correspond to an enrichment of the reference class among genes that are downregulated in the high CD204 expression group. Upregulated and downregulated related gene sets were enriched in the KEGG and HALLMARK pathways, respectively. Significant gene sets were affirmed when the absolute score of NES was ≥1.0, NOM P-val was ≤0.05, and FDR q-val was ≤0.25.

### Evaluation of tumor-infiltrating immune cells in different CD204 mRNA expression status

The CIBERSOR algorithm was used to calculate the subdivided immune cell types recognized as LM22 among the groups with high and low CD204 mRNA expression groups split by the median value in the different subtypes [57].

### Correlation analysis of CD204 and immunoinhibitors

The online tool TISIDB and R2 database (https://hgserver1.amc.nl) were used to perform correlation analyses of CD204 mRNA and immunomodulators from the TCGA database among the luminal A and B, HER-2-enriched, and basal-like breast cancer subtypes [58]. Pearson correlation analysis was used.

### Cell culture and reagents

Human MDA-MB-231, T47D, MCF7, SKBR3 and THP-1 cell lines were all obtained from Cell Bank of the Chinese Academy of Sciences (Shanghai, China) and passed the test of DNA profiling (short tandem repeat (STR) profiling method). All these breast cancer (BC) cells (MDA-MB-231, T47D, MCF7 and SKBR3) were cultured following the instructions of the American Type Culture Collection (ATCC, Manassas, VA, USA). For M2 tumor-associated macrophages (M2-TAMs) generation, as previously described, THP-1 cells were first treated with 200 nM Phorbol-12-myristate-13-acetate (PMA) (Sigma-Aldrich, USA) for 24 h, then PMA-THP-1 cells (polarized into macrophages) were cultured by the addition of conditioned media from BC cells (MDA-MB-231, T47D, MCF7 and SKBR3) for another 48 h. In a co-culture system, THP-1 cells were differentiated in upper chamber (0.4 μm pore) (Corning, USA), and BC cells (MDA-MB-231, T47D, MCF7 and SKBR3) were seeded in the lower chamber. After 48 h, TAMs or BC cells were harvested for further study.

### Western blotting

The antibodies for western blotting in our study included anti-β-actin (Abways, Shanghai, China) and anti-CD204 (Abcam, Cambridge, UK).

Western blotting was performed as previously described [59]. The total protein extraction buffer (Beyotime, Shanghai, China) was used follow the manufacturer's instructions. Further, equal amounts of proteins were separated by SDS-PAGE gel electrophoresis and transferred onto a polyvinylidene fluoride (PVDF) membrane. After blocking with 5% skimmed milk, the membrane was probed with the following primary antibodies: anti-CD204 (1:1000) and anti-β-actin (1:2000). The species-matched secondary antibodies were used (1:10000), and the bands were detected by the Odyssey imaging system (LI-COR, Lincoln, NE, USA).

### Plasmid construction and transfection

The plasmid containing full-length CD204 and a negative control plasmid were obtained from FulenGen Ltd., Co. (Guangzhou, China). TAMs were transfected with CD204 and negative control plasmids using Lipofectamine LTX and PLUS regents (Invitrogen, USA) follow the manufacturer’s instructions. After 72 h, the efficacy of CD204 overexpression was tested by Western blotting.

### CCK-8 and colony formation assays

CCK-8 assay was carried out according to the manufacturer's instructions (Dojindo Molecular Technologies, Japan). In brief, target cells (3 × 10^3^) were seeded in 96-well plates. The optical absorbance at 450 nm was detected using a plate reader (Thermo Fisher Scientific) at different time points (0, 24, 48, 72, 96, and 120 h).

For the colony formation assay, as previously described, the target cells (5 × 10^3^) were cultured per well of a 6-well plate for 2 weeks. Then, colonies were fixed with paraformaldehyde and stained with 0.5% (w/v) crystal violet. Photographs were acquired and the cell numbers were counted [59].

### Wound healing, transwell migration and invasion assays

For wound healing assay, the cells were grown to a confluent monolayer, following which a line was scratched through the monolayer in each well and non-serum media was added. Three representative wound lines were selected for measurement. The wound areas were visualized and measured at 0 and 24 h post-wounding.

In the migration assay, 5 × 10^4^ cells were seeded into the upper chamber of the transwell plate (Millipore, La Jolla, CA, USA). The cell invasion assay was performed using Matrigel-coated filters (BD, La Jolla, CA, USA). Specific culture medium was added to the bottom chamber. As previously described, BC cells were allowed to migrate for 24 h or invade through the Matrigel for 48 h [60]. The migrated or invaded cells were then fixed and stained with 0.1% crystal violet, six randomly selected fields were photographed, and the cell numbers were counted.

### Statistical analyses

Data are presented as the mean ± standard deviation (SD). Statistical analyses were conducted using GraphPad Prism 5 software. Comparisons between the groups were performed using two-tailed Student’s t-test. Values of p < 0.05 were considered to be statistically significant.

## Supplementary Material

Supplementary Figure 1
