# Supplementary material for: Clinical and transcriptional signatures of human CD204 reveal an applicable marker for the protumor phenotype of tumor-associated macrophages in breast cancer
Source: Aging (Albany NY). 2019 Dec 4;11(23):10883–901. doi: 10.18632/aging.102490 (PMC6932883; doi:10.18632/aging.102490)
Supplement: Supplementary Figure 1 [file aging-11-102490-s001..pdf]

SUPPLEMENTARY FIGURE

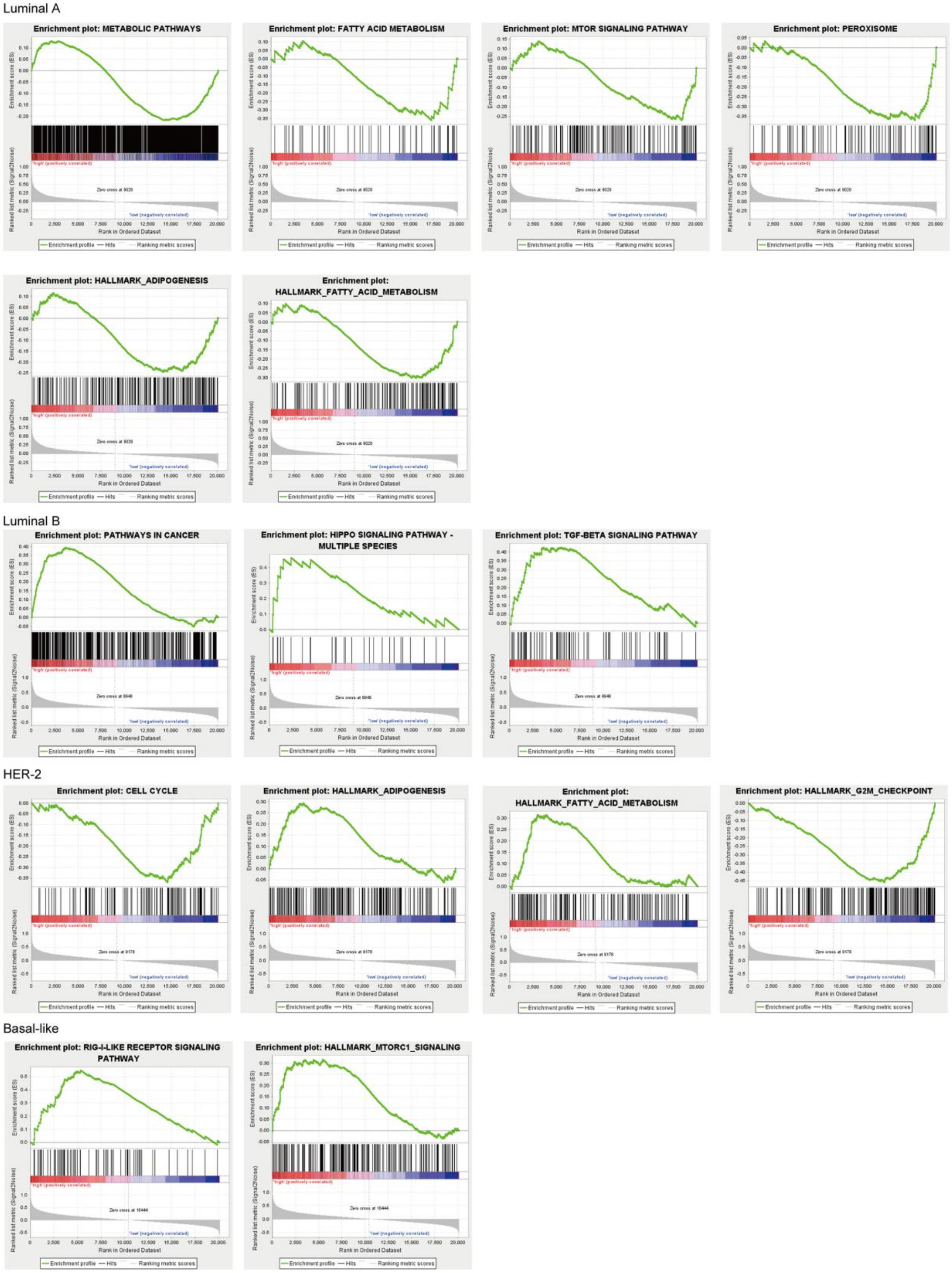

Supplementary Figure 1. Detailed and representative special pathways in each subtype influenced by CD204.
